# Design and Machine Learning Modeling of a Multi-Degree-of-Freedom Bionic Pneumatic Soft Actuator

**DOI:** 10.3390/biomimetics10090615

**Published:** 2025-09-12

**Authors:** Yu Zhang, Linghui Peng, Wenchuan Zhao, Ning Wang, Zheng Zhang

**Affiliations:** College of Mechanical Engineering, Shenyang University of Technology, Shenyang 110870, China; zhangyu@sut.edu.cn (Y.Z.); wenchuanzhao@sut.edu.cn (W.Z.); 17624040725@163.com (N.W.); zzhang_1119@163.com (Z.Z.)

**Keywords:** soft pneumatic actuator, multi-degree of freedom, bionic sea turtle, inertial measurement unit, machine learning modeling

## Abstract

A novel multi-degree-of-freedom bionic Soft Pneumatic Actuator (SPA) inspired by the shoulder joint of a sea turtle is proposed. The SPA is mainly composed of a combination of oblique chamber actuator units capable of omnidirectional bending and bi-directional twisting, which can restore the multi-modal motions of a sea turtle’s flipper limb in three-dimensional space. To address the nonlinear behavior of the complex structure of SPA, traditional modeling is difficult. The attitude information of each axis of the actuator is extracted in real time using a high-precision Inertial Measurement Unit (IMU), and the attitude outputs of the SPA are modeled using six machine learning methods. The results show that the XGBoost model performs best in attitude modeling. Its R^2^ can reach 0.974, and the average absolute errors of angles in Roll, Pitch, and Yaw axes are 1.315°, 1.543°, and 1.048°, respectively. The multi-axis attitude of the SPA can be predicted with high accuracy in real time. The studies on deformation capability, actuation output performance, and underwater validation experiments demonstrate that the SPA meets the bionic sea turtle shoulder joint requirements. This study provides a new theoretical foundation and technical path for the development, control, and bionic application of complex multi-degree-of-freedom SPA systems.

## 1. Introduction

With the continuous breakthroughs in the field of bionic robotics, researchers are paying more and more attention to the resolution and reproduction of complex motion capabilities of living organisms. Sea turtles have become an important reference object for underwater bionic robot design due to their efficient propulsion and excellent environmental adaptability demonstrated in underwater motion. In particular, its shoulder joint, through coordinating complex multi-degree-of-freedom actuation, realizes wide-range, supple, and flexible paddling movements, which is an important inspiration for underwater robots to enhance propulsion efficiency and mobility [1,2].

Traditional rigid joints have limitations such as limited motion flexibility and insufficient safety in interaction with the environment. To overcome these problems, soft robots have gradually become a cutting-edge hotspot in recent years. By adopting bionic structures and materials, soft robots have good deformation ability and environmental adaptability, and have a wide range of applications in high safety and smooth operation scenarios [3,4]. According to the actuation method, they can be categorized into many types, such as electric, thermal, magnetic, optical, and fluid actuators [5,6,7,8,9]. Among them, Pneumatic Soft Actuator (SPA) is widely used in medical assistance, industrial grasping, and bionic robotics due to its lightweight, high flexibility, large deformation, and good bionic properties [10,11,12]. Conventional SPAs are mostly single-degree-of-freedom single-chamber or tandem modular designs, which are challenging to meet the demand of complex spatial motion. Researchers try to realize multi-degree-of-freedom output from the structural level, such as a single oblique chamber structure [13,14] and a combined chamber [15,16,17], to further enhance the spatial coupling and control capability. In recent years, multi-degree-of-freedom bionic design concepts have been introduced into soft-body structures, such as Huang et al. proposed a novel bionic omnidirectional bending soft-body actuator (BOBA) inspired by mollusks such as leeches and caterpillars, etc. The BOBA possesses the characteristics of small radial expansion, fast response speed, good flexibility, and omnidirectional bending, etc. [18]. Multi-degree-of-freedom SPA inspired by a turtle was designed to realize the gait motion of a quadrupedal pneumatic crawling robot by adjusting the air pressure in different chambers [19]. The octopus-inspired design of a multi-degree-of-freedom soft-body robotic arm utilizes linear air chambers to achieve bending and elongation motions, and helical chambers to achieve bi-directional twisting motions [20]. These advances have greatly expanded the functional boundaries of SPAs, but there is a lack of design and mechanical modeling toward a multi-degree-of-freedom, compact SPA that can be analogous to the shoulder joint of a sea turtle.

To achieve the predictability and controllability of multi-degree-of-freedom actuators, modeling is essential. The main modeling approaches include: Force or moment balance methods: These decompose the expansion force within the chambers into component forces acting on the structure, deriving mechanical equations that relate chamber pressure to deformation [21,22]. This method is also used in the mechanical modeling of bistable soft structures [23]. Principle of virtual work: this approach constructs force–displacement relationships by minimizing the system’s total energy and is a typical modeling method for soft actuators [24,25]. Finite element method (FEM): FEM is employed to precisely model how chamber deformation and material distribution affect motion performance and mechanical behavior [26,27]. It is also used for motion modeling in multi-material reinforced structures [28]. Some studies have also developed composite deformation models that simultaneously account for torsion, bending, and elongation under geometrical orthotropic conditions and nonlinear elasticity [29,30]. Due to the high degree of flexibility and strong nonlinear characteristics of soft-bodied robots themselves, their motion processes are highly susceptible to the effects of their own gravity and external perturbations. These perturbations are often difficult to model and predict, especially in unstructured environments [31]. To address the problem, researchers have gradually turned to model-free modeling. Model-free modeling does not require explicit knowledge of the internal mechanism of the system. However, it dynamically uses learning and iteration to adjust the modeling model by observing the response relationship between inputs and outputs [32]. Machine learning (ML) methods have been gradually introduced into SPA modeling in recent years. A nonlinear mapping function is constructed by collecting input air pressure, output, and attitude or position data. Standard methods include: regression models: linear regression (LR), support vector machine (SVR), random forest regression (RFR), gradient boosted tree regression (GBR), XGBoost regression (XGBR), decision tree regressor (DTR), and k-nearest neighbor (KNN), etc.; neural network models: multilayer perceptron (MLP), convolutional network (CNN), and recurrent network (LSTM), etc.; Deep Reinforcement Learning: for online learning and motion planning, especially for interactive tasks [33,34]. Thuruthel et al. targeted a homemade soft robotic finger by embedding soft sensors inside it and combining it with a laser motion capture system to synchronize the acquisition of barometric pressure, sensor data, and end position. A neural network was used to model the motion of the soft body finger using the air pressure and sensor values as inputs and the end coordinates as outputs. The model accuracy was verified [35]. Ye et al. implemented a modeling method that combines the finite element method and multilevel analysis, laying the foundation for the control and practical application of the soft fiber-reinforced bending actuator [36]. Youssef et al. modeled the soft pneumatic actuator with different motion angles using an echo state network (ESN) to deal with irregular time sequence data. The ESN model successfully predicted the actuator end position by training with experimental data and optimizing hyperparameters, demonstrating its potential in dealing with nonlinear complex systems [37]. The above modeling approaches and recent research advances provide a theoretical basis for the controllability and predictability of SPA and expand the possibilities for its application in complex real-world environments. Since there are no mature models that can effectively describe the complex dynamic behavior of our designed multi-degree-of-freedom bionic sea turtle shoulder joint SPA. Therefore, this study focuses on developing an accurate and robust modeling framework for such systems using model-free learning methods.

Based on the above background, this paper designs and prepares a new type of multi-degree-of-freedom pneumatic SPA with the shoulder joint of a bionic sea turtle as the research object. The structural characteristics and motion mechanism of SPA are systematically analyzed, and its force and deformation characteristics are studied. Combined with the experimentally collected end attitude motion data, the dynamic modeling of the SPA is carried out using machine learning methods to realize the modeling and intelligent prediction of the soft bionic joint system. Finally, its motion and mechanical properties are analyzed, and the feasibility of SPA as a bionic turtle shoulder joint is verified by underwater experiments. This study not only provides a theoretical basis for the control of multi-degree-of-freedom SPA, but also lays a foundation for the design and application of underwater bionic robots.

## 2. Materials and Methods

### 2.1. Structural Design

**Figure 1:**
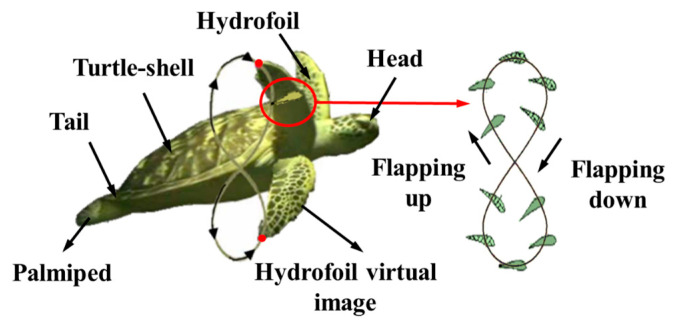
Introduction diagram of the parts of a sea turtle and a sketch of its motion.

A sketch of sea turtle motion is shown in Figure 1 [38]. The flipper motion is the primary power source of the turtle’s movement, with a negative head-on angle (with the incoming velocity pointing toward the positive airfoil surface) and a positive head-on angle to the relative incoming current during the flapping up and flapping down phases, respectively. Both phases of motion are horizontally driven and generate thrust, which is accompanied by lift, which causes the body to generate vertical acceleration, the magnitude of which depends on the angle-of-approach distribution. The motion of the hydrofoil tip relative to the turtle shell is in the shape of a thin “8”. Therefore, the end of the shoulder joint needs to be bent around the x–axis and y–axis, as well as twisting around the z–axis.

**Figure 2:**
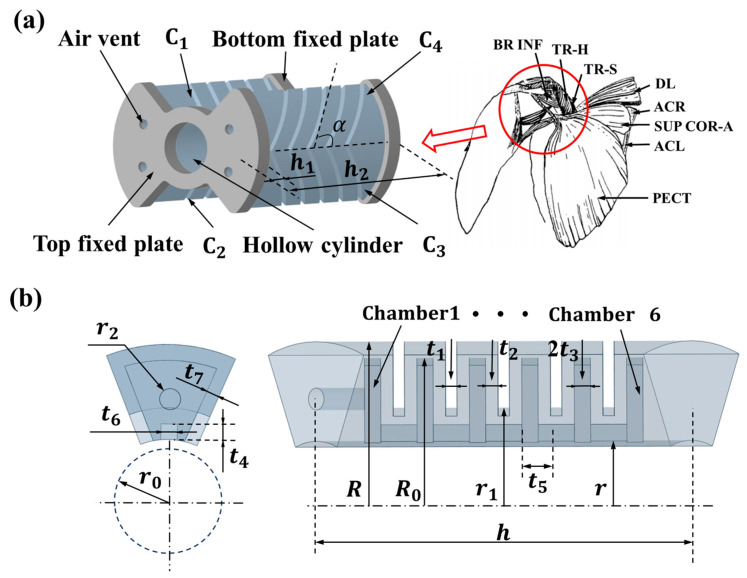
Schematic diagram of the SPA structure and dimensions of the bionic sea turtle’s shoulder joint. (**a**) The structure diagram of SPA corresponds to the muscles of the sea turtle shoulder joint. (**b**) Details and parameters of an oblique chamber actuator unit.

The sea turtle’s upper arm motion is driven by the shoulder muscles, the names of the various parts of which are shown on the right side of Figure 2a. The abbreviated and full names of the muscles are shown in Table 1. They enable flipper flexion and extension, as well as the forward and backward motion of the forelimb. Adduction and Abduction— movements that move the limb closer to and away from the midline of the body. Rotation—the rotational motion of the shoulder joint around its own axis, including internal and external rotation [39].

Inspired by the biology of sea turtles, the structural diagram of our designed bionic sea turtle shoulder joint SPA is shown in Figure 2a. The SPA mainly consists of four oblique chamber actuator units ($C_{1}$, $C_{2}$, $C_{3}$, and $C_{4}$), a hollow cylindrical structure composed of a non-retractable layer, and top and bottom fixation plates. Its oblique chamber angle is α=45°, outer radius R=64 mm, total height $h_{2}=100$ mm, and inner radius $r_{0}=14$ mm. The structure and dimensions of the SPA oblique chamber actuator units are given schematically in Figure 2b, where one end of the oblique chamber actuator units contains a vent hole, and the internal chambers are linearly arranged along the axial direction of the actuator, and the chambers are interconnected with each other through air channels. Among them, the top and bottom fixing plates are fixedly connected to the four oblique chamber actuator units and the hollow cylindrical structure, which are intended to keep the hollow cylinders from being squeezed to ensure the overall deformation effect of the SPA. The parameter details of each part are shown in Table 2.

### 2.2. Fabrication

**Figure 3:**
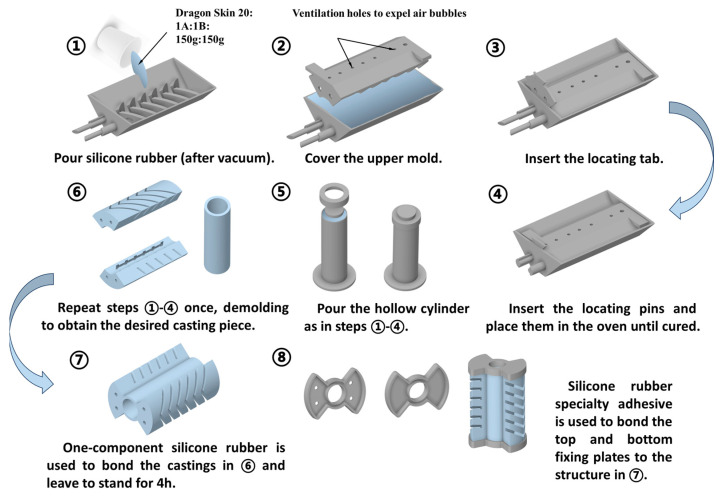
Process flow diagram for SPA pouring and bonding.

The SPA is manufactured in the following steps:

1. Design and 3D print the required mold, top, and bottom fixing plates. Apply petroleum jelly evenly on the surface of the mold for subsequent demolding.

2. The specific casting and bonding process is shown in Figure 3 below:

① Take equal amounts of Dragon Skin 20 silicone rubber AB components (150 g each, mass ratio 1:1) and mix thoroughly. Pump under vacuum for 10 min to remove air and reduce air bubbles during pouring. Slowly pour the mixture into the mold until it is filled.

② Slowly press into the upper mold to fit the non-perforated end.

③ Insert the locating tab, block locating pin holes by hand, and pull out the locating pins.

④ Insert the locating pin and place the whole in the oven at 70 °C until the silicone elastomer is cured.

⑤ Pour the hollow cylinder as in steps ① to ④.

⑥ Repeat steps ① to ④ and then demold to get the desired casting.

⑦ Use one-component silicone rubber JD-851 to bond the demolded castable and leave it to cure for 4 h.

⑧ Use the solid Baili 9955 quick-drying adhesive to bond the adhesive parts obtained in step ⑦ with the top and bottom fixing plates, and leave it for 4 h, and finally combine it to form the SPA. All the reinforcement structures are made by 3D printing with PLA resin.

3. Four sections of air tubes of suitable length are inserted and firmly bonded to the vent tube interface for subsequent air supply and pressurization.

This fabrication method enables rapid manufacturing of the SPA. Subsequent experimental results show that the actuator is manufactured with high repeatability, high quality, and excellent performance.

### 2.3. Experimental Platforms and Data Acquisition

**Figure 4:**
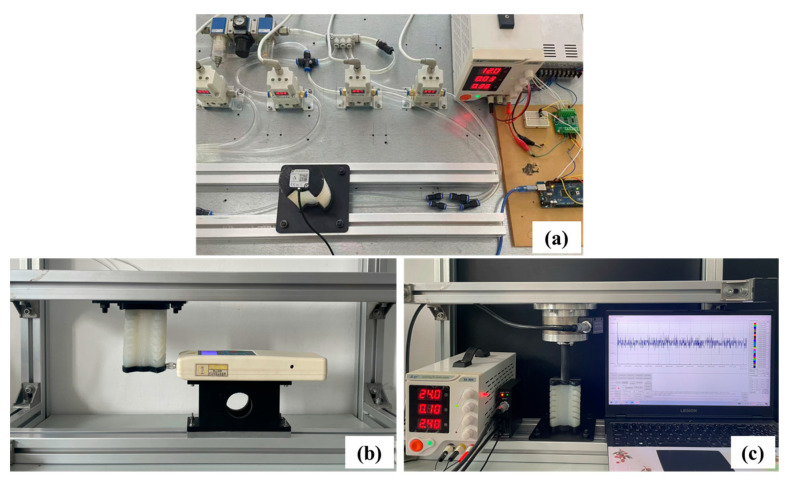
Experimental platform for SPA pneumatic control, data acquisition, and measurement of bending force and torque. (**a**) Experimental platform for the SPA pneumatic control and data acquisition. (**b**) Bending force measurement. (**c**) Torque measurement.

As shown in Figure 4a, the pneumatic control experiment platform is constructed. First, the host computer generates and sends control commands to the controller, and the electrical signals generated by the controller are transmitted to the signal conversion module and the relay, respectively. The signal conversion module converts the received electrical signals into corresponding voltage signals. The electric proportional valve adjusts the valve opening according to the voltage signal, thus realizing the precise control of the air pressure. Furthermore, the opening and closing sequence, as well as the driving pressure of each chamber of the SPA, can be regulated accordingly.

The output performance of a SPA is usually evaluated by the force and moment that can be generated at its end, for which a specialized test setup is built. As shown in Figure 4b, to test the mechanical output of the SPA under different air pressures, the end of the SPA is tightly connected to a force gauge. The input pressure is gradually adjusted, and the change in the force value under each pressure is recorded. To obtain the torque data, another set of measurement schemes, as shown in Figure 4c, is used. In the experiment, the SPA is coupled to a six-dimensional force-torque transducer via a solid shaft to transfer the generated torque to the transducer. The sensor is connected to the M8128 data acquisition device via the Remo interface. Then, it communicates with the host computer using the serial line to the RJ45 interface and the acquisition device to provide the required 24 V power supply. Ultimately, dedicated software monitors the collected analog data in real time.

As shown in Figure 4a, an IMU sensor (FDI DETA10-PW) is fixedly mounted at the end of the SPA to realize the real-time monitoring of motion status. Its accuracy, drift effect, sampling frequency, and repeatability all meet the subsequent attitude data acquisition, change trend extraction, and machine learning model training requirements.

The selection of input pneumatic pressure data should specifically include the following cases:

1. Only one chamber is inflated;

2. Two chambers are inflated with equal pressure;

3. Two chambers are inflated with unequal pressures;

4. Three chambers are inflated with equal pressure;

5. Three chambers are inflated with different pressures;

6. All four chambers are inflated with equal pressure;

7. All four chambers are inflated with different pressures.

The input air pressure of each way is in the range of 0–120 kPa. Following the above air pressure selection, to obtain a stable output angle value, it is kept for 5s after inflation and for 5 s after restoring the original position in the inflation. The acquisition frequency is 20 HZ. Considering the drift accumulation and zero drift of the IMU in long-term acquisition, this paper adopts the segmented mean difference method to process the raw attitude data. A certain number of trapezoidal data sets are obtained under different pressure combinations. The specific processing method of data filtering and selection: the data are divided into multiple intervals by timestamp segmentation, and outlier filtering is performed on each data segment (based on the standard deviation or percentile calculation of the threshold). The mean difference between neighboring segments is then calculated to extract dynamically changing features. Segmentation captures the phase characteristics of the time series, outlier filtering reduces noise interference, and mean-value difference computation highlights the trend of interval changes. The overall method is flexible and efficient, suitable for analyzing the nonlinear characteristics of dynamic systems, and at the same time improves the data quality and reliability of the results.

### 2.4. Machine Learning Methods

#### 2.4.1. Database Construction and Data Preprocessing

Eight hundred data sets have been obtained through data acquisition and processing using the experimental platform described above. To avoid significant numerical differences among the feature input parameters and to improve the efficiency and accuracy of machine learning (ML) modeling, it is generally necessary to normalize and standardize the data before training ML models.

Performing normalization first eliminates the extreme impact of outliers by bringing all features to a unified scale, and subsequent standardization further enhances the regularity of the data distribution. This approach prevents adverse effects during model training caused by significant feature dimension or distribution differences. It increases data suitability, enabling most machine learning models to capture the essential relationships between features accurately. The formula for standardization is as follows:

(1) $$z=\frac{x_{i}-\mu }{\sigma }$$

where z denotes the eigenvalue of the i-th sample after standardization, $x_{i}$ is the eigenvalue of the i-th sample before standardization, μ is the mean eigenvalue of all samples, and σ is the standard deviation of the eigenvalue of all samples.

#### 2.4.2. Algorithm Introduction

Based on the Anaconda platform, six machine learning algorithms are implemented in Spyder’s integrated development environment using the built-in Scikit-learn library. Each of these algorithms has advantages and is suitable for different data modeling tasks.

In this study, model-free modeling of the multi-degree-of-freedom SPA is formulated as a regression problem rather than a classification problem. To identify the optimal algorithm for modeling, six ML algorithms are compared, covering linear models (LR), distance-based k-nearest neighbor methods (KNN), kernel-based methods (SVR), and ensemble tree methods (including RFR, GBR, and XGBoost), to perform a comparative analysis for data-driven prediction of the actuator’s motion angles. The dataset is divided into two disjoint sets: the training and testing sets. The training set size is 75%, while the testing set accounts for 25%. During model optimization, Monte Carlo cross-validation is employed.

#### 2.4.3. Algorithm Evaluation

To quantify the prediction accuracy of the models, the following two types of evaluation metrics are employed: (1) Mean Absolute Error (MAE), which is the average of the absolute errors between the predicted results and the actual observed values across all experiments; (2) the coefficient of determination (R^2^), which characterizes the fitting ability of the predictions to the actual data. The calculation formulas for both metrics are as follows:

(2) $$MAE=\frac{1}{N}\sum_{i=1}^{N}\left|\theta_{i}^{a}-\theta_{i}^{p}\right|$$

(3) $$R^{2}=1-\frac{{\sum_{i=1}^{N}\left(\theta_{i}^{a}-\theta_{i}^{p}\right)}^{2}}{{\sum_{i=1}^{N}\left(\theta_{i}^{a}-\bar{\theta_{i}^{a}}\right)}^{2}}$$

where $\theta_{i}^{a}$ and $\theta_{i}^{p}$ denote the actual and predicted values of the motion angle of the SPA, respectively. $\bar{\theta_{i}^{a}}$ denotes the average value of the actual motion angle of the SPA, and N denotes the number of instances. The angle value can be expressed as the angle value under the motion around the x, y, and z axes, respectively.

## 3. Results and Discussion

First, the SPA is analyzed in terms of force and motion patterns to clarify the superposition and offset relationship of the axial forces under different inflation states, as well as the specific deformation patterns generated under the action of these forces. Subsequently, six machine learning models were used to model the SPA, and the modeling effects of each model were compared and analyzed to select the optimal model. Finally, the deformation capacity and braking output performance of the SPA are summarized, and its practical application value is verified through application scenarios.

### 3.1. Force and Motion Mode Analysis

**Figure 5:**
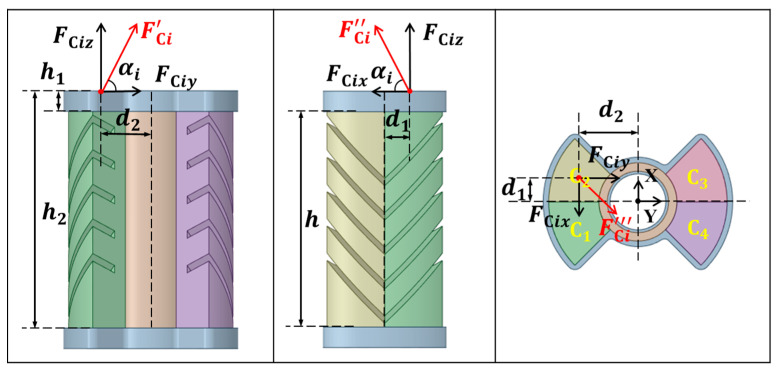
Force analysis diagram of the SPA.

When either diagonal chamber actuator is driven by pressurization, a direct force will be exerted on the hollow cylindrical structure of the confining layer. In order to systematically analyze the force and deformation response of this structure under different chamber aeration conditions, and to gain a deeper understanding of the changes in force distribution when each chamber is inflated synergistically or individually, this paper firstly conducts force and motion deformation analyses for the case of a dual-chamber synchronous filling with the same air pressure. Subsequently, on this basis, the structural deformation characteristics of single chamber inflation and more complex multi-chamber combined driving cases are further explored to comprehensively reveal the effects of different driving modes of the actuator unit on the mechanical properties of SPA. Figure 5 shows the force analysis of the SPA. The combined forces in the corresponding directions generated by one of the oblique chamber actuator units are shown in Figure 5 denoted as $F_{Ci}^{\prime }$, $F_{Ci}^{\prime \prime }$, and $F_{Ci}^{\prime \prime \prime }$, respectively.

Two chambers inflated simultaneously with equal pressure: When actuator units on the same side are inflated simultaneously, force components with equal magnitude and opposite direction in the structure cancel each other. For example, when chambers $C_{1}$ and $C_{2}$ are inflated, the forces generated by $F_{C1x}$ and $F_{C2x}$ have equal magnitude and opposite direction. The two x-axis direction forces, $F_{C1y}$ and $F_{C2y}$, add up and cause the SPA to rotate clockwise around the x–axis. Similarly, with $C_{1}$ and $C_{4}$ chambers, $F_{C1y}$ and $F_{C4y}$ also have equal magnitudes and opposite directions, so the two y–axis direction forces, $F_{C1x}$ and $F_{C4x}$, add up and bend the SPA counterclockwise around the y–axis. Therefore, when the oblique chamber actuator units on the same side are activated, part of the force acts in the opposite direction and is absorbed, making the SPA rotate around the x–axis or y–axis, referred to as Bending-x and Bending-y, respectively. Likewise, when actuator chambers arranged diagonally (such as $C_{1}$ and $C_{3}$) are inflated at the same time, the directions of the force components are opposite and the line connecting their points of application is not parallel to the x–axis or y–axis, which produces a net torque around the z–axis and causes the SPA to generate Twisting-z. The top fixed plate and bottom connecting plate are designed to reinforce the hollow cylinder, enabling stable rotation while avoiding deformation of the internal central cylinder.

Single chamber inflated: When only one chamber actuator is inflated, the SPA ordinarily shows a combined motion around the x–axis, y–axis, and z–axis. The force caused by pneumatic expansion acts directly on the layer-hollow cylinder wall and is decomposed into orthogonal components along the x–axis and y–axis. Together, these component forces contribute to the bending of the SPA, and the specific bending direction is determined by the position of the activated chamber and the direction of the corresponding force. In addition, since the aerodynamic forces usually act off the center axis of the hollow cylinder, a certain moment is formed in the structure, triggering a certain twisting of the SPA.

Multiple chambers inflated simultaneously: When multiple chambers are inflated at the same time, the resultant forces are added or offset as vectors on the surface of the cylinder wall. The specific motion mode is determined by the magnitude and direction of the component forces. If the force components in a certain direction are fully balanced, the SPA does not show macroscopic displacement in that direction. If they are unbalanced, the SPA is driven to bend. Furthermore, when the chamber pressures are distributed asymmetrically, and the line connecting the force application points is not parallel to the x–axis or y–axis, the combined force forms a net torque along the z–axis.

The correspondence between each axial motion of the SPA described above and the way the oblique chamber actuator units are inflated is detailed in Table 3. It is worth noting that the oblique chamber angle α is a key parameter for achieving multi-degree-of-freedom motion in the SPA, and its change directly affects the final movement pattern. Both bending and twisting usually involve energy loss and partial cancellation of the force components, resulting in reduced actuation energy efficiency. Thus, the efficiency changes as α varies. Especially when α=90°, the forces generated by the symmetric chambers tend to cancel each other out, so only bending around the x–axis and y–axis remains, and the twisting effect is essentially suppressed. Previous studies indicate that an SPA with α=45° most closely matches the motion pattern of a sea turtle’s shoulder joint [40].

### 3.2. Machine Learning for Model-Free Modeling

**Figure 6:**
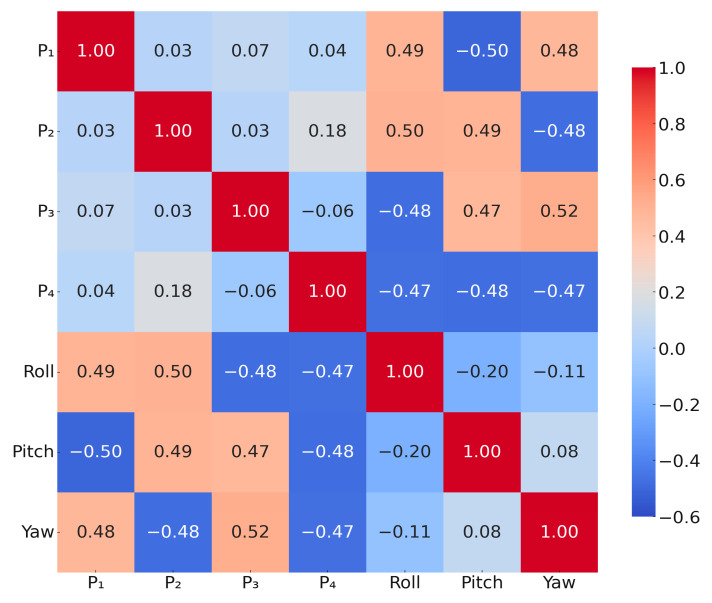
Correlation heatmap between two of the main features in the data.

The linear correlation between the seven variables is effectively portrayed in Figure 6, corresponding to the above-mentioned pair of charging cases corresponding to force deformation. Roll corresponds to the motion around x–axis described earlier, positively correlating with $C_{1}$ and $C_{2}$, and negatively correlating with $C_{3}$ and $C_{4}$; pitch corresponds to the motion around the y–axis, positively correlating with $C_{2}$ and $C_{3}$, and negatively correlating with $C_{1}$ and $C_{4}$; Yaw corresponds to the motion around the z–axis, positively correlating with $C_{1}$ and $C_{3}$, and negatively correlating with $C_{1}$ and $C_{4}$; motion positively correlated with $C_{1}$ and $C_{3}$, and negatively correlated with $C_{2}$ and $C_{4}$. These correlation results are consistent with the main motion modes and the response characteristics of each sensor after the structure is inflated, indicating that the feature selection is physically reasonable. In addition, most of the absolute values of the correlation coefficients are lower than 0.5, and no significant high covariance is observed, indicating that there is no obvious redundant relationship among these seven features, and, thus, they are suitable for use as input variables for subsequent machine learning modeling.

**Figure 7:**
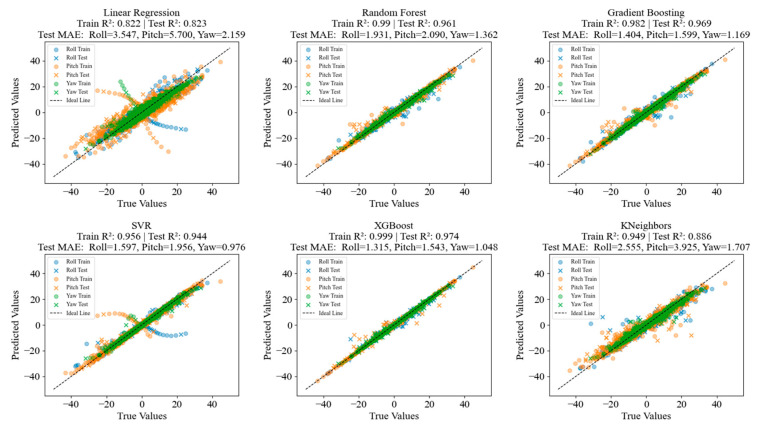
Comparison of regression prediction performance of multiple models for Roll, Pitch, and Yaw.

As shown in Figure 7, the six regression models (LR, RFR, GBR, SVR, XGBoost, and KNN) exhibit significant differences in predictive performance for the regression tasks of Roll, Pitch, and Yaw angles. In each subplot, the horizontal axis represents the true values and the vertical axis denotes the predicted values, with ideal fit points expected to be evenly distributed around the dashed line. The performance comparison is as follows: the test set R^2^ of LR is 0.823, which is clearly inferior to the other nonlinear models. The test set mean absolute errors (MAE) for Roll, Pitch, and Yaw are 3.547, 5.700, and 2.159, respectively, with the largest error occurring in the Pitch direction. A large number of points are scattered on both sides of the ideal line, and the prediction error increases significantly where the target values are more dispersed, indicating that LR fails to capture the complex nonlinear relationships in the data. The three ensemble methods (RFR, GBR, and XGBoost) demonstrate strong fitting and generalization abilities. Their R^2^ values on the test set all exceed 0.96, which is substantially better than those of LR and KNN, and their MAEs in all angular directions are notably lower than those of other single nonlinear models. Among them, XGBoost is particularly outstanding, achieving a test set R^2^ of 0.974 and MAE values of 1.315, 1.543, and 1.048 for Roll, Pitch, and Yaw, respectively—the best scores among all models—which reflects its effective balance between model complexity and generalization performance. In contrast, SVR achieves a test set R^2^ of 0.944, better than LR and close to the ensemble methods, but its errors in the Pitch and Roll directions are slightly higher. The plots also show somewhat more scattered points, especially in the Pitch direction, indicating that SVR’s generalization to extreme samples is somewhat inferior to that of ensemble models. KNN exhibits the lowest performance among all nonlinear models, with a test set R^2^ of 0.886. The MAE values indicate that its predictive accuracy is less than that of the ensemble methods and SVR, and its predictions are more sensitive to the distribution of nearest samples. The point clouds are more scattered, particularly with a significant increase in errors in the Pitch direction, which suggests that its capability to fit high-dimensional continuous variables is limited.

In summary, nonlinear ensemble methods (such as XGBoost, RFR, and GBR) perform best in the regression prediction tasks for pose angles, significantly outperforming LR and KNN. Among these, XGBoost, which integrates various optimization strategies, achieves the highest prediction accuracy and the lowest MAE, making it the optimal choice for this task.

**Figure 8:**
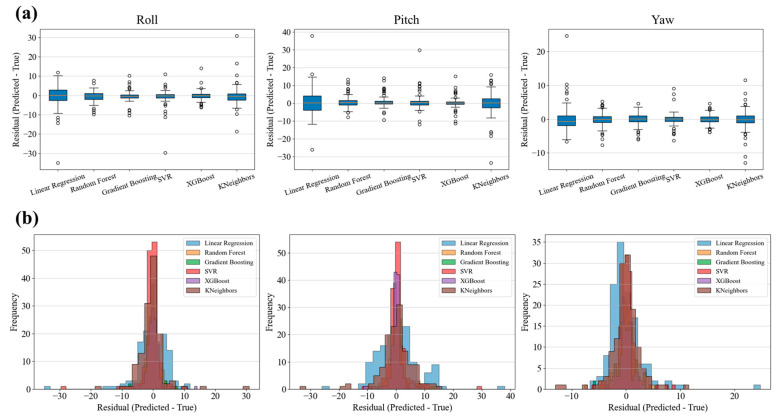
Box plots and histograms of prediction errors for each angular direction for different models. (**a**) Box plots of errors; (**b**) Histograms of error distributions.

As shown in Figure 8 by the residual box plots and distribution histograms, the ensemble models, such as XGBoost and GBR, exhibit much more concentrated prediction error distributions on all three attitude angles (Roll, Pitch, Yaw), with fewer extreme errors and outliers, indicating better generalization performance. In contrast, LR and KNN display wider residual distributions and more extreme points in all motion angles. This is consistent with the previously discussed evaluation metrics (such as R^2^ and MAE), further confirming the superiority of ensemble learning models.

The developed machine learning models achieve highly accurate mapping between pneumatic inputs and attitude outputs. Building upon this prediction capability, the deformation capability and actuation output performances of the multi-degree-of-freedom SPA are further investigated.

### 3.3. Analysis of Deformation Capability and Actuation Output Performance

To evaluate the motion capability of the multi-degree-of-freedom SPA under different ventilation modes, various air pressure combination modes listed in Table 3 are selected for experimental measurement and comparative analysis. The results show that the maximum bending angle around the x–axis can reach ±65.504°, ±50.6° around the y–axis, and the twisting angle around the z–axis is ±52.5°. The maximum pressure-bearing capacity is 120 kPa; exceeding this value may lead to chamber expansion and instability. When equal pressure is applied to all four chambers, the actuator achieves a maximum elongation deformation of 9.4 mm, without significant twisting or bending. The deformation capability is sufficient to meet the requirements of a biomimetic turtle shoulder joint.

**Figure 9:**
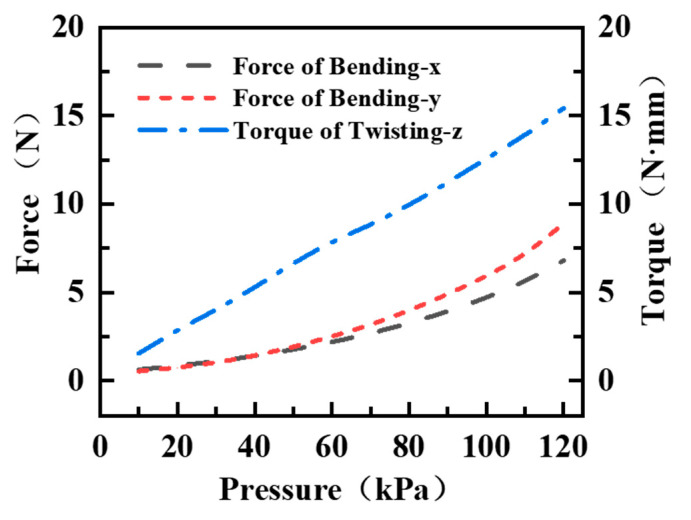
Curves of bending force and torque as a function of pressure under the same air pressure in both chambers of the SPA.

In addition, since the force comes from multiple asymmetric chambers, the resulting bending and twisting variations are driven by both the combined force and the combined torque, which exhibits nonlinear coupling characteristics between different combination modes. Using the force and torque experimental platform shown in Figure 4b,c, the maximum force and torque under pressure change can be obtained when the same air pressure is passed into the two chambers, as shown in Figure 9 below. It can be seen that the maximum force and torque increase with increasing pressure. The maximum force is 6.81N and 8.86N for Bending-x and Bending-y, and the maximum torque is 15.43 N · mm.

**Figure 10:**
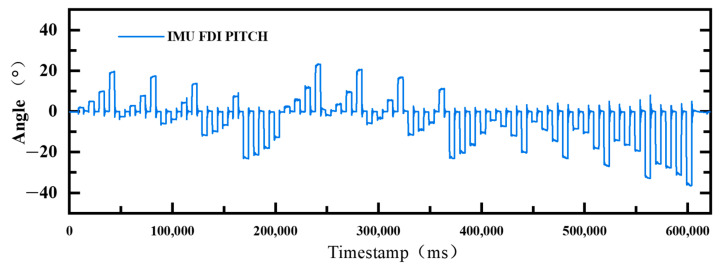
Real-time angular variation of the PITCH angle measured by the IMU when the two chambers of the SPA are inflated with different pressures.

By processing all the experimental data and selecting some of the experimental data representative of the two cavities subjected to different pressures in Figure 10 below for description. The horizontal coordinate is the time in milliseconds, and the vertical coordinate is the pitch angle. The figure shows that each climb during inflation is almost a vertical line, which changes rapidly and generally increases slightly with increasing pressure. The deflation process is equally steep and sometimes steeper than inflation. It can be concluded that deflation tends to be faster than inflation. Similarly, it can be seen that inflation reaches a state after a certain angle faster than deflation reaches stability. The analysis of the experimental data shows that the hysteresis and response characteristics of the SPA can meet the dynamic requirements of the bionic turtle shoulder joint well.

### 3.4. Performance Validation of the SPA

**Figure 11:**
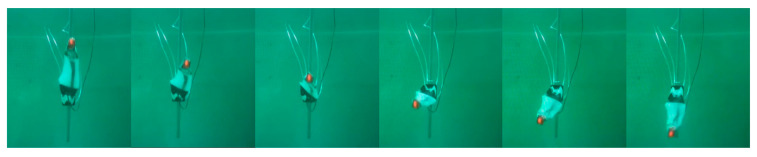
Half-cycle diagram of flipper motion of the bionic sea turtle.

In order to further verify the actual operational performance of the pneumatic flexible bionic actuator, it is attached to the flipper for underwater drive performance verification, and an experimental test system is constructed, as shown in Figure 11. The schematic diagram of the motion of the bionic sea turtle flipper during half a cycle is shown in the figure. It is verified that the SPA, as a bionic shoulder joint, can execute the corresponding motion of the flipper limb and reach the corresponding angle through modeling. The binocular camera recognizes its end position. Therefore, SPA provides design inspiration and references for developing bionic sea turtle robots.

## 4. Conclusions and Outlook

This study presents an innovative design of a compact multi-degree-of-freedom oblique chambers SPA with omnidirectional bending and bidirectional twisting capabilities for a biomimetic turtle shoulder joint robot. The proposed SPA simulates the complex spatial movements of a turtle’s shoulder joint in underwater environments, providing new insights and technological foundations for the miniaturization and modularization of underwater flexible actuation modules.

Through experiments, 800 sets of pneumatic input and attitude output data are collected under single- and multi-chamber ventilation conditions. A data processing method based on timestamp segmentation and mean-difference-based IMU drift suppression is proposed, effectively ensuring the stability and reliability of attitude measurements. Considering the characteristics of multiple inputs and outputs, high nonlinearity, and strong coupling, a forward modeling framework from pneumatic input to attitude output is established, and six mainstream machine learning methods are systematically compared. The results demonstrate that the ensemble learning model XGBoost performs best on roll, pitch, and yaw axis attitude prediction tasks, significantly outperforming other algorithms. The model exhibits good generalization and deployment efficiency, making it suitable for embedded and online control scenarios. The actuator’s deformation capability and actuation output performance are comprehensively analyzed. The results indicate that under an ultimate pressure of 120 kPa, the maximum bending and twisting angles of the SPA around the x, y, and z axes reach ±65.5°, ±50.6°, and ±52.5°, respectively, with a maximum axial elongation of 9.4 mm. The maximum bending forces around the x– and y–axes are 6.81 N and 8.86 N, while the maximum torque around the z–axis is 15.43 N·mm, fully meeting the requirements of a biomimetic turtle shoulder joint. Subsequent underwater verification experiments further confirm these findings.

In summary, the proposed multi-DOF SPA and its high-precision modeling approach solve the nonlinear mapping problem of complex biomimetic joint motions, providing model-free modeling support and application examples for the design and intelligent control of underwater flexible robots with high-dimensional input-output systems. This method can be extended to various biomimetic scenarios such as underwater propulsion, flexible fingers, and exoskeletons, thereby expanding the engineering application boundaries of soft robotics.

However, this work has some limitations. For example, temporal features are not included in the modeling, which may affect trajectory tracking and dynamic control performance; nor is the underwater environment’s nonlinear influence on the shoulder joint’s terminal attitude considered. Future research will delve deeper and broaden the current study, focusing on the following directions: developing end-to-end inverse control based on the existing modeling framework; integrating closed-loop control and sensor fusion for precise feedback and stable control; and extending the model to control validation under underwater binocular vision recognition and external disturbances.

## Figures and Tables

**Table 1:** The abbreviated and full names of the muscles of sea turtle shoulder joint.

Abbreviated Name	Full Name	Abbreviated Name	Full Name
BR INF	Brachialis	ACR	Acromion
TR-H	Triceps humeral h.	SUP COR-A	Supracoracoideus anterior part
TR-S	Deltoideus ventral part	ACL	Acromial-coracoid ligament
DL	Deltoideus ventral part	PECT	Pectoralis major

**Table 2:** The parameter details of the SPA.

Parameters		Parameters	
α	45°	$r_{1}$	20
n	6	$t_{1}$	2.9
h	84	$t_{2}$	3.182
$h_{1}$	8	$2t_{3}$	4.15
$h_{2}$	100	$t_{4}$	3.021
R	32	$t_{5}$	9.264
$R_{0}$	29	$t_{6}$	3.116
r	14	$t_{7}$	2.5
$r_{0}$	11	$t_{8}$	3

**Table 3:** Inflation and motion mode fact sheet of the SPA.

Inflatable Chambers Number	Is the Pressure the Same		Motion Mode	Axis	Inflatable Chambers
One chamber			Multi-axis motion	+x, +y, +z	Take $C_{1}$ as an example
	
Two chambers	Same		Bending-x	+x	$C_{1}{+C}_{2}$
				−x	$C_{3}{+C}_{4}$
			Bending-y	+y	$C_{2}{+C}_{3}$
				−y	$C_{1}{+C}_{4}$
			Twisting-z	+z	$C_{1}{+C}_{3}$
				−z	$C_{2}{+C}_{4}$
	Different		Multi-axis motion	x, y, z	Any two chambers
Three chambers	Same		2-axis or multi-axis motion	x, y/x, z/y, z x, y, z	Any three chambers
	Different		2-axis or multi-axis motion	x, y/x, z/y, z x, y, z	
Four chambers	Same		Extensor motion	+z	$C_{1}{+C}_{2}{+C}_{3}{+C}_{4}$
	Different		Single-axis or two-axis or multi-axis motion	x/y/z x, y/x, z/y, z x, y, z	$C_{1}{+C}_{2}{+C}_{3}{+C}_{4}$

## Data Availability

The data supporting this study’s findings are available from the first author or corresponding author upon reasonable request.
